# The Case of X and Y Localization of Nucleolus Organizer Regions (NORs) in *Tragulus javanicus* (Cetartiodactyla, Mammalia)

**DOI:** 10.3390/genes9060312

**Published:** 2018-06-20

**Authors:** Anastasia A. Proskuryakova, Anastasia I. Kulemzina, Polina L. Perelman, Natalia A. Serdukova, Oliver A. Ryder, Alexander S. Graphodatsky

**Affiliations:** 1 Institute of Molecular and Cellular Biology, SB RAS, Lavrentiev ave 8/2, Novosibirsk 630090, Russia; zakal@mcb.nsc.ru (A.I.K.); polina.perelman@gmail.com (P.L.P.); serd@mcb.nsc.ru (N.A.S.); graf@mcb.nsc.ru (A.S.G.); 2 Novosibirsk State University, Pirogova str. 1, Novosibirsk 630090, Russia; 3 San Diego Zoo Institute for Conservation Research, San Pasqual Valley Rd 15600, Escondido, CA 92027, USA; oryder@sandiegozoo.org

**Keywords:** karyotype, chromosome evolution, genome, chevrotain, sex chromosomes, ribosomal DNA

## Abstract

There are differences in number and localization of nucleolus organizer regions (NORs) in genomes. In mammalian genomes, NORs are located on autosomes, which are often situated on short arms of acrocentric chromosomes and more rarely in telomeric, pericentromeric, or interstitial regions. In this work, we report the unique case of active NORs located on gonоsomes of a eutherian mammal, the Javan mouse-deer (*Tragulus javanicus*). We have investigated the position of NORs by FISH experiments with ribosomal DNA (rDNA) sequences (18S, 5.8S, and 28S) and show the presence of a single NOR site on the X and Y chromosomes. The NOR is localized interstitially on the p-arm of the X chromosome in close proximity with prominent C-positive heterochromatin blocks and in the pericentromeric area of mostly heterochromatic Y. The NOR sites are active on both the X and Y chromosomes in the studied individual and surrounded by GC enriched heterochromatin. We hypothesize that the surrounding heterochromatin might have played a role in the transfer of NORs from autosomes to sex chromosomes during the karyotype evolution of the Javan mouse-deer.

## 1. Introduction

Nucleolus organizer regions (NORs) are crucial for the formation of the nucleolus and contain multiple copies of the ribosomal clusters interdigitated with non-transcribed intergenic spacers [1]. This ribosomal DNA encodes 45S pre-rRNA. The post-transcription processing results in the production of three types of ribosomal RNA (18S, 5.8S, and 28S) [2]. The ribosomal RNA is further processed and packed into large (5.8S, 28S, and separate non-nucleolus 5S rDNA) and small (18S) subunits of the ribosome, that are responsible for RNA translation and thus critical for cellular functions [1]. These ribosomal DNA sequences exhibit a high degree of conservation across animals. In contrast with sequence conservation, rDNA clusters are characterized by variation in copy-number [3], rDNA content of NORs between individuals [4], between the cells of one individual [5], and variable localization in different animal species [6,7]. The variation in the number of transcriptionally active NORs can occur even between the cells and tissues of one individual [8]. NORs are predominantly located in telomere regions, colocalizing with telomeric repeats [9], in the short arms or near centromeric regions of acrocentric chromosomes [10,11,12,13] and more rarely interstitially [14,15]. The quantity of ribosomal genes at telomeric sites is variable [16,17]. In most mammalian species, NORs are situated on autosomes. However, localization of NORs on sex chromosomes is not uncommon in fish [18,19,20,21] and insects [12,14].

There is a problem in mammals with a localization of NORs on sex chromosomes, due to heterogametic sex and dosage compensation, and only a few cases have been described. In some marsupials, the NOR located on the X seems to escape X-inactivation [22,23]. Thus, specific mechanisms exist for regulating NOR expression in these cases. The localization of a NOR on the X was shown in Seba’s short-tailed bat (*Carollia perspicillata*) [24]. An inactive NOR was described for *Lemniscomys barbarus* (Muridae, Rodentia) X and Y chromosomes and its participation in the sex chromosome recombination process was suggested [25]. Thus, localization of rDNA clusters on sex chromosomes in mammals is extremely rare.

The mammalian order Cetartiodactyla provides a few examples of sex chromosome localization of NORs. In the Indian muntjac (*Muntiacus muntjac*) (Cervidae) NORs are localized on the sex chromosomes due to the translocation of a NOR-carrying autosome onto the X chromosome [6,26]. The male nilgai (*Boselaphus tragocamelus*) (Bovidae) is another example of sex-chromosome localization of a NOR. However, there is a complex sex chromosome structure consisting of a translocated autosome 14 onto the X and Y, and the NOR is located on the 14 part of compound 14;X but not on 14;Y [15]. We have not found in literature other examples of sex chromosome localization of NORs in Cetartiodactyla.

In this study, we describe the sex chromosome localization of a NOR in a Javan mouse-deer karyotype (Tragulidae), provide its molecular cytogenetic characterization by localization of ribosomal genes, and investigate the activity of these NORs and surrounding regions.

## 2. Materials and Methods

### 2.1. Cell Culture and Chromosome Preparation 

Metaphase chromosomes were obtained from cultured fibroblast cell line. Cells were incubated at 37 °C and 5% CO_2_ in αMEM (Gibco, Paisley, Scotland, UK), supplemented with 15% fetal bovine serum (Gibco), 5% AmnioMAX-II (Gibco) and antibiotics (ampicillin 100 μg/mL, penicillin 100 μg/mL). Metaphases were obtained by adding colcemid (0.01 μg/mL) and EtBr (0.05 μg/mL) to actively dividing culture [27]. Hypotonic treatment was performed with KCl (33 mM) and trisodium citrate (7.7 mM) for 20 min at 37 °C and followed by fixation with 3:1 methanol-glacial acetic acid fixative. Metaphase chromosome preparations were made from fixed cultures, as described previously [28].

### 2.2. Nucleolus Organizer Regions Detection

To reveal the localization of NORs, plasmid DNA (pHr13 [29]), containing human partial 18S, full 5.8S, part of the 28S ribosomal genes and two internal transcribed spacers, was amplified with a GenomePlex Whole Genome Amplification kit (Sigma-Aldrich Co., St. Louis, MO, USA) and labeled with biotin (Roche, Basel, Switzerland) by GenomePlex WGA Reamplification Kit (Sigma-Aldrich Co., St. Louis, MO, USA). The banding pattern obtained by standard trypsin/Giemsa (GTG) treatment of metaphase chromosomes was photographed prior to fluorescence in situ hybridization (FISH) [30]. FISH was performed as described by F. Yang and A. Graphodatsky [28].

### 2.3. Silver Staining of Metaphase Chromosome

To reveal active NORs the silver staining of metaphase chromosome with AgNO_3_ was performed according to [31]. 

### 2.4. The Combined Method of Heterogeneous Heterochromatin Detection (CDAG)

Heterochromatin analysis was performed by the Combined Method of Heterogeneous Heterochromatin Detection (CDAG) method [32]. AT- and GC-enriched repetitive sequences were detected by DAPI (4′-6-diamidino-2-phenylindol) and CMA3 (chromomycin A3) fluorescent dyes. All images were captured and processed using Video-test 2.0 Image Analysis System and a Baumer Optronics CCD Camera mounted on an Olympus BX53 microscope (Olympus, Shinjuku, Japan).

## 3. Results

**Figure 1:**
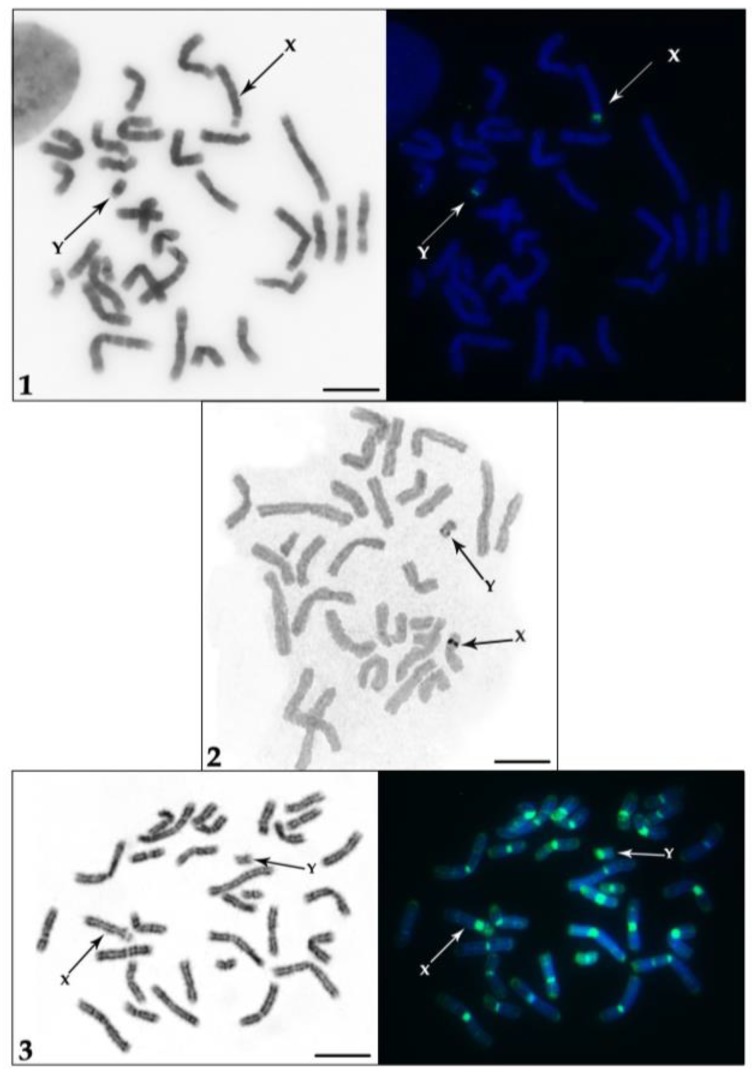
Metaphase chromosomes of *Tragulus javanicus*: (**1**) Localization of 18S, 5.8S, and 28S ribosomal DNA (rDNA): inverted DAPI (4′-6-diamidino-2-phenylindol)-staining (**left**) and labeled DNA sequences (**right**), (**2**) AgNO_3_ staining of the Nucleolus organizer regions (NORs), (**3**) CDAG staining: GTG-banding (**left**) and CMA3/DAPI-staining (**right**). Scale bar = 10 µm.

In order to obtain data about active and silent NORs, we have performed AgNO_3_ staining and localized rDNA sequences (18S, 5.8S and 28S, which represent main part of the ribosomal cluster) by FISH (Figure 1(1)), respectively. We have identified the only pair of active NORs (Figure 1(2)) on sex chromosomes. Interestingly this NOR is located not only on the X but also on the subtelocentric Y chromosome. The NOR on the X chromosome is larger and positioned in the middle of the p-arm at a secondary constriction. On the Y chromosome, it is located close to the centromere.

To investigate chromosomal regions of the NORs and to describe the adjacent heterochromatin we performed consecutive differential staining: a GTG-banding to identify chromosomes followed by CDAG-staining to uncover AT- and GC-rich heterochromatin (Figure 1(3)). We observed that pericentromeric heterochromatin on the X and the Y is comprised of both AT- and GC-rich sequences. The interstitial heterochromatic block on the X chromosome is composed of GC-rich sequences. On the X there are two prominent C-positive bands [33] on both sides of the NOR—the proximal one is GC-rich and the distal one is slightly GC-enriched. The adjacent heterochromatin band on the q-arm of the Y is also GC-rich. Notably, both *T. javanicus* NORs are CMA3 positive proving support that these regions are enriched in GC sequences [29].

## 4. Discussion

Nucleolus organizer regions play an important role in genome functioning and are prominent cytogenetic markers. Among mammals, there are just a few cases of the NOR localization on sex chromosomes [6,15,26,34]. We have found a unique case of NOR localization on both X and Y chromosomes in the Javan mouse-deer. This case shows the importance of the use of multiple chromosome staining techniques while describing karyotype of species to reveal features of genome organization on chromosome level.

**Figure 2:**
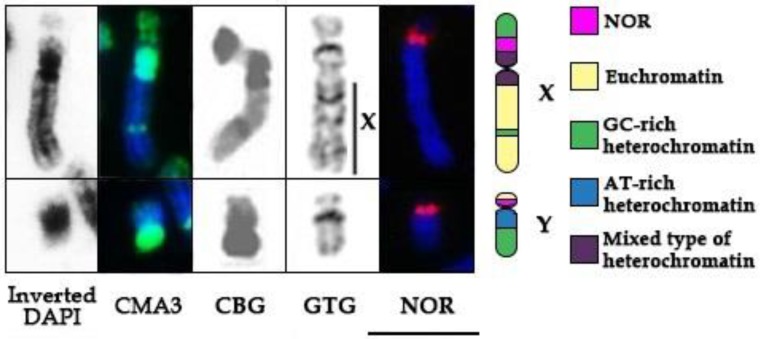
Differential staining of sex chromosomes (X, Y) of Javan mouse-deer: Inverted DAPI (AT-rich heterochromatin) CDAG-staining; CMA3 (GC-rich heterochromatin) CDAG-staining; CBG (CBG–C-bands by Barium hydroxide using Giemsa) [33]; GTG (G-bands by Trypsin using Giemsa) [35]; DAPI-staining and FISH localization of 18S, 5,8S and 28S rDNA probe. Scale bar = 10 µm.

The Javan mouse-deer possesses a karyotype with 2n = 32, NF = 64 with all bi-armed chromosomes. G-, C- and Q-banded karyotypes of Javan mouse-deer were described by D. S. Gallagher with co-authors [33]. C-banding revealed prominent heterochromatin blocks covering almost the entire p-arm of the X and proximal part of the q-arm and one interstitial block in the distal part of the q-arm (Figure 2). The Y chromosome is mostly composed of a large heterochromatin block. The probe containing 18S + 5.8S + 28S rDNA covers most of the active ribosomal cluster. Multiple copies of this cluster form the nucleolus organizer region demonstrate a distinct localization of NORs near the centromere region on the X and Y chromosomes in the middle of large heterochromatic blocks. The CDAG staining indicates that the NORs in *T. javanicus* are located in the proximity of GC-rich heterochromatin region.

It remains unexplained how the NOR that is typically located on the autosomes in Cetartiodactyla was translocated onto the sex chromosomes. There are data about NOR variation in the number and chromosomal location in closely related species, suggesting that rDNA clusters are highly mobile components of the genome [4,36]. In species related to Javan mouse deer, such as gray whale [13] and giraffe [37] NORs are situated on non-homologous chromosomes representing different syntenic groups. Such interspecies lability of NORs is generated either by chromosomal rearrangements or transposition events [38]. Possibly, the high transposition rates of NORs are observed because of transposons inserted in the rDNA clusters, as it has been documented in plants [39] and into intergenic spacer repeats in the house mouse [40]. However, the transposition of NORs from autosome to sex chromosome may also be due to the translocation of an ancestral autosome carrying NORs. The rare cases of the addition of autosomal material to the sex chromosomes have been reported previously [41], including one with the NOR translocation in the nilgai bull [15]. The clustered NOR structure also might contribute to its instability, facilitated by an illegitimate recombination between non-homologous chromosomes. It results in variation of the number of sequences per NOR and the localization on various chromosomes even in closely related species [7].

The Javan mouse-deer X chromosome [42] and karyotype [35] were formed as a result of many changes during cetartiodactyl genome evolution. Telomeres and centromeres in the Javan mouse-deer karyotype have prominent C-banded regions as well as interstitial C-bands. Ribosomal gene sequences might migrate with the surrounding heterochromatin onto sex chromosomes during genome rearrangements, particularly to the X chromosome, followed by subsequent fixation. It may also be possible that the adjacent abundant heterochromatin facilitated NOR transfer during recombination [43]. The NOR in a species of May bug (*Melolontha melolontha*) is surrounded by heterochromatin and exhibits the main features of fragile sites such as gaps, deletions and chromatid exchanges [14]. The NORs in *Tragulus javanicus* is also surrounded by heterochromatin and may be prone to fragile site behavior.

It also remains unknown whether this positioning of the NOR provided a selective advantage for the species. In meiosis, there is a recombination block in NORs, heterochromatic, and highly repeated sequences [25,44]. But in *Lemniscomys barbarus* (Muridae, Rodentia) [25] the NOR sequences translocated onto sex chromosomes promoted the increase in the recombination region. In this species, X-Y pairing proceeds beyond the pseudoautosomal region colocalizing with the silent ribosomal sequences and heterochromatin [25]. Such an increase of the recombination region may lead to new genetic variants providing selective advantages, leading to a fixation of the NORs on the sex chromosomes. It would be useful to study the X-Y recombination region in Javan mouse-deer because the NORs sequences are adjacent to heterochromatic elements situated in these regions, like in *L. barbarus* [25].

It would be interesting to investigate whether the NOR is adjacent to the pseudoautosomal region (PAR) on both the X and the Y chromosome in Javan mouse-deer, as well as to inspect the NOR localization in other chevrotain species. Unfortunately, there is no whole genome sequence data or chromosome assembly for Tragulidae species available to design a Y chromosome PAR probe. Comparative cattle BAC mapping data indicate that PAR in *Tragulus* is likely to be situated on the q-arm of X chromosome immediately distal to the heterochromatic block [41]. Chromosome painting indicates that the euchromatin portion occupies only the distal part of the q-arm of the X [42], so it is possible that the NOR and the pseudoautosomal region are separated by a large heterochromatin block on the X. However, the location of the PAR on the *T. javanicus* Y chromosome is unknown. It is possible that the NOR and the PAR on the Javan mouse-deer Y chromosome are positioned similarly to the dog Y where the PAR and the NOR are separated by the centromeric heterochromatin block [45], but we cannot exclude p-arm localization of the PAR.

The sex chromosome (both the X and the Y) localization of the single active NOR surrounded by GC-rich heterochromatin in Javan mouse-deer represents a unique case among mammals and warrants further investigation of the link between ribosomal clusters and heterochromatin and also of the poorly understood role of NOR variation in karyotype and species evolution processes.
